# The impact of high-frequency rTMS treatment on brain activity in PSCI patients: a TMS-EEG study

**DOI:** 10.3389/fneur.2025.1582437

**Published:** 2025-05-14

**Authors:** Xinxin Song, Jianming Fu, Yunhai Yao, Yuhong Shu, Zhongli Wang, Xuting Chen, Lianjie Ma, Fang Shen, Xiaolin Sun, Xiaoqing Ma, Ting Zhang, Rujue Jin, Ming Zeng, Xudong Gu

**Affiliations:** ^1^Joint Training Base of Zhejiang Chinese Medical University and Jiaxing University, Hangzhou, China; ^2^Rehabilitation Medicine Center, Second Affiliated Hospital of Jiaxing University, Jiaxing, China

**Keywords:** post-stroke cognitive impairment, repetitive transcranial magnetic stimulation, TEPS, TMS-EEG, EEG functional connectivity

## Abstract

**Objectives:**

This study employed Transcranial Magnetic Stimulation combined with Electroencephalography (TMS-EEG) to examine the impacts of high-frequency repetitive transcranial magnetic stimulation (rTMS) on brain activity and cognitive function in patients with post-stroke cognitive impairment (PSCI), focusing on changes in connectivity of the left dorsolateral prefrontal cortex (DLPFC) across different frequency bands.

**Methods:**

Twenty subacute PSCI patients were recruited for a 20-day rTMS treatment, consisting of 10 days of sham stimulation followed by 10 days of actual stimulation. Clinical function scale data and TMS-EEG data were collected before treatment (Pre), after sham stimulation (Sham), and after rTMS treatment (TMS) to analyze transcranial magnetic stimulation evoked potentials (TEP), time-frequency, and functional connectivity. Additionally, a *post hoc* subgroup analysis was conducted to assess the impact of education level, time since onset, and lesion size on cognitive score improvement.

**Results:**

Compared to the Pre and Sham conditions, cognitive function and daily living ability scores significantly improved post-rTMS. Although the TEP patterns in the Pre and Sham conditions were similar, rTMS enhanced the early TEP amplitude in the left DLPFC, slowed gamma oscillations, increased connectivity in the theta and alpha bands in the bilateral DLPFC, and altered the connectivity patterns between the left DLPFC and other brain regions. Changes in theta-band wPLI were significantly positively correlated with improvements in MMSE scores (*r* = 0.465, *p* = 0.039) and MoCA scores (*r* = 0.493, *p* = 0.027). Patients with higher education levels exhibited significant cognitive improvement (*p* = 0.039), while patients with a time since onset of 60–180 days showed a significant decline in cognitive improvement (*p* = 0.024).

**Conclusion:**

High-frequency rTMS effectively modulated connectivity patterns between the left DLPFC and other brain regions in PSCI patients, enhancing cognitive functions. Changes in wPLI within the theta frequency band may serve as a potential biomarker for cognitive function improvement in PSCI patients. Education level and time since onset may have a certain impact on cognitive improvement in PSCI patients.

## 1 Introduction

Post-stroke cognitive impairment (PSCI) is primarily characterized by varying degrees of impairment in one or more cognitive domains, including memory, attention, executive function, visuospatial abilities, orientation, and language skills, and may be accompanied by psychological and behavioral symptoms (1). The incidence of post-stroke cognitive impairment is relatively high, with approximately 83% of patients demonstrating cognitive decline in at least one cognitive domain after a stroke (2). This significantly hinders their functional recovery and severely impacts their quality of life and longevity (3). Consequently, cognitive impairment has become a major burden for stroke patients and has garnered significant attention in stroke research and clinical intervention.

Repetitive transcranial magnetic stimulation (rTMS) is a non-invasive cortical stimulation technique designed to regulate the activity of specific brain regions through repeated stimulations and frequency adjustments. Depending on the stimulation parameters, rTMS can either inhibit or excite the targeted brain areas, promoting neural plasticity and the reorganization of brain function, thereby improving cognitive function (4). The left dorsolateral prefrontal cortex (DLPFC) is involved in various higher cognitive functions and is closely related to cognitive impairment in patients with PSCI (5). The long-term potentiation (LTP) effects produced by high-frequency rTMS stimulation of the left DLPFC depend on glutamatergic neurotransmission and are modulated by cholinergic, dopaminergic, and GABAergic systems (6).

Simultaneous recording of transcranial magnetic stimulation and electroencephalography (TMS-EEG) is an innovative method that can capture the summation of post-synaptic excitatory and inhibitory potentials in response to TMS pulses, known as TMS-evoked potentials (TEPs) (7). TMS-EEG demonstrates high reproducibility, allowing repeated evaluations after 1 week, and is highly sensitive to changes in stimulation intensity, location, and angle (8). This technique can be used to assess changes in cortical excitability and connectivity after stroke, and TMS-EEG can monitor dynamic changes in brain function following a stroke, providing potential biomarkers for predicting cognitive recovery (9). TEPs have been applied to studies of various cognitive states and connectivity characteristics. The most commonly observed peaks in TEPs of the left DLPFC include P30, N45, P60, N100, P180, and N280. Early peaks primarily reflect specific activity of the left DLPFC, while later peaks show lower dependence on it (10, 11). TMS pulses can trigger signal propagation between cortical regions. Compared with resting-state EEG connectivity, TMS-evoked functional connectivity has a higher signal-to-noise ratio and helps to reveal the causal relationships between different cortical regions (12, 13).

Although previous studies have shown that high-frequency rTMS stimulation of the left DLPFC can improve cognitive function in PSCI patients (14–16), and have explored the TEP changes in the left DLPFC of healthy individuals (17), the specific mechanisms by which rTMS improves cognitive function in PSCI patients through TMS-EEG remain unclear. To further elucidate these mechanisms, this study conducted an in-depth analysis of TEP and functional connectivity in PSCI patients.

## 2 Materials and methods

### 2.1 Participants

A case-control study was conducted from November 2023 to December 2024 at the Second Hospital of Jiaxing City, Zhejiang Province. The study included 20 patients with post-stroke cognitive impairment who received management of underlying conditions (such as hypertension, diabetes, etc.) and conventional rehabilitation therapies (including comprehensive training for hemiplegic limbs, occupational therapy, etc.). Further details are provided in Table 1.

**Table 1:** Clinical characteristics of stroke patients.

Participants	Age (years)	Gender	Education (years)	Lesion location	Time since onset (days)	RMT	MMSE	MoCA	MBI
sub01	49	M	12	R: Basal ganglia ischemic	69	37	24	23	44
sub02	52	F	18	L: Fronto-parietal lobe hemorrhagic	24	35	21	19	63
sub03	47	M	12	R: Basal ganglia hemorrhagic	74	55	25	23	66
sub04	75	M	15	R: Frontal-temporal, insular basal ganglia ischemic	78	34	25	24	50
sub05	60	M	12	L: Pontine ischemic	34	26	20	19	52
sub06	58	M	12	R: Lateral ventricle ischemic	132	35	25	23	56
sub07	61	M	6	R: Frontal lobe hemorrhagic	82	28	24	22	55
sub08	65	M	6	L: Basal ganglia brainstem ischemic	24	54	19	20	38
sub09	45	M	16	L: Basal ganglia ischemic	56	35	18	19	77
sub10	71	F	0	R: Brainstem ischemic	18	35	20	20	39
sub11	53	M	11	R: Thalamic ischemic	19	37	24	23	44
sub12	56	M	6	R: Basal ganglia thalamus hemorrhagic	23	25	23	22	30
sub13	62	F	12	R: Basal ganglia subarachnoid hemorrhagic	29	30	19	19	55
sub14	70	F	6	L: Frontal-temporal lobe hemorrhagic	41	54	17	18	35
sub15	72	M	3	R: Frontal lobe ischemic	50	40	22	26	40
sub16	49	M	9	L: Occipital lobe ischemic	30	42	20	25	49
sub17	55	M	2	R: Parietal lobe ischemic	45	38	19	24	34
sub18	58	M	6	L: Basal ganglia ischemic	20	36	18	23	42
sub19	63	M	5	R: Temporal lobe hemorrhagic	15	31	23	27	58
sub20	70	F	0	L: Brainstem ischemic	36	29	21	25	37

M, Male; F, Female; L, Left; R, Right.

Eligible participants were screened using the Montreal Cognitive Assessment (MoCA) and their cognitive function was assessed with the Mini-Mental State Examination (MMSE) scale (18, 19). The modified Barthel index (MBI) was used to evaluate the patients’ activities of daily living. In this study, we have obtained permission to use the MMSE and MoCA scales. All participants refrained from psychotropic drugs, caffeine, and alcohol, and were right-handed. They met the exclusion criteria established by international TMS safety standards. The study was approved by the Ethics Committee of the Second Hospital of Jiaxing University (No. jxey-202403301) and conducted in accordance with the ethical principles outlined in the Declaration of Helsinki. It was registered with the Chinese Clinical Trial Registry (Registration No. ChiCTR2400090135), all participants signed a written informed consent form and voluntarily participated in the transcranial magnetic stimulation treatment. The inclusion criteria for stroke patients were: (1) age between 30 and 75 years, (2) MoCA score <26 (20), (3) the stroke criteria are consistent with the latest diagnostic standards (21), (4) time since onset between 2 weeks and 6 months, (5) good language comprehension and expression abilities, able to actively cooperate with treatment. Exclusion criteria included: (1) other neuropsychiatric disorders, such as Alzheimer’s disease, Parkinson’s disease, or a history of substance abuse, (2) cognitive impairment due to other causes, (3) epilepsy or history of intracranial surgery that presents contraindications for rTMS, (4) inability to cooperate due to cognitive or language impairments, (5) presence of severe somatic diseases or intracranial diseases.

### 2.2 TMS-EEG recording

Before the treatment, motor evoked potentials (MEPs) were measured in the left primary motor cortex (M1) region of the subjects. The stimulation intensity was set to the resting motor threshold of the first dorsal interosseous muscle, with a maximum stimulator output of 100% (22). This required that at least 5 out of 10 consecutive stimulations elicited MEPs with an amplitude of at least 50 μV (23). It is known that in some stroke patients, MEPs cannot be elicited even at maximum stimulator output (24). If no MEPs can be detected in the damaged left hemisphere, the threshold of the right hemisphere should be used to guide the treatment intensity (25).

TMS-EEG was recorded using a 64-channel TMS-compatible EEG system (NeuroHub wearable multimodal research platform, Neuracle, China). Electrodes were placed according to the international 10–20 system using Ag/AgCl 64 electrodes. Single-pulse transcranial magnetic stimulation (TMS) was applied to the left dorsolateral prefrontal cortex after determining the coil position with the EEG cap. The coil should be placed tangentially to the scalp at the position of the AF3 electrode on the electrode cap, with the handle pointing backward at an angle of approximately 45° to the midline. Single-pulse transcranial magnetic stimulation (TMS) at a frequency of 1 Hz was applied at the AF3 electrode using 100% resting motor threshold, with each stimulation lasting for 1 s, an interval of 4 s between stimulations, and a total duration of 9 min, consisting of 108 pulses. EEG was recorded from the 64 electrodes (with REF as the online reference and GND as the ground electrode) using an average reference, sampling from 0.1 to 1,000 Hz while keeping the impedance between the scalp and electrodes below 5 kΩ. To reduce eye movements, participants were instructed to fixate on a black cross on a white background approximately 2 meters away (26). Noise-canceling headphones were used to play white noise, preventing the subjects from hearing the sound of the coil stimulation (below 90 dB) (27).

### 2.3 Repetitive transcranial magnetic stimulation

**Figure 1:**
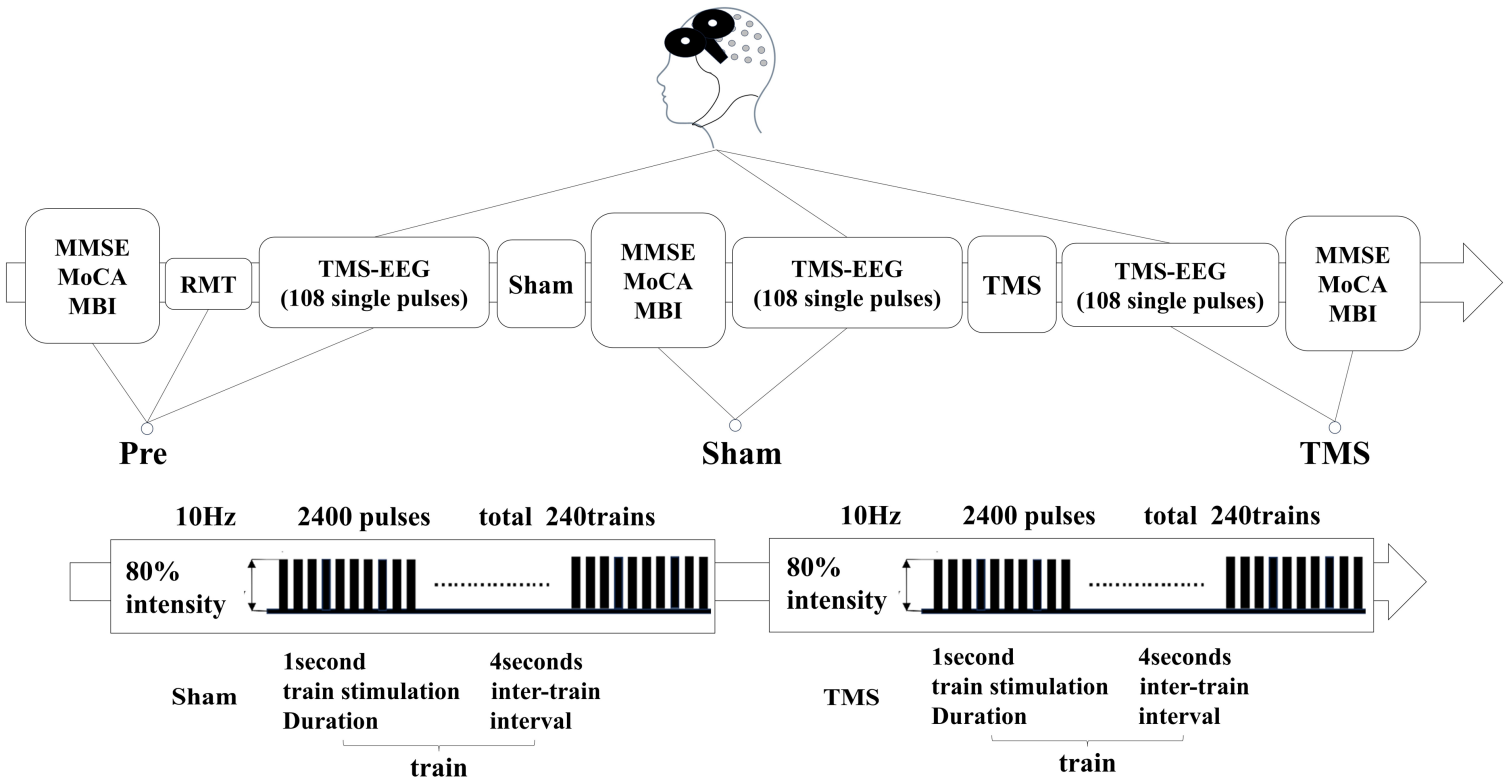
In patients with PSCI, the resting motor threshold (RMT) was measured before treatment. They underwent three TMS-EEG sessions and scale assessments at the Pre, Sham, and TMS time points. The second column represents the rTMS stimulation sequences for both sham and real stimulation.

rTMS (RT-50, Junjian Wanfeng, China) was administered at an intensity of 80% to the left DLPFC. The rTMS protocol consisted of delivering 10 sequences of 1-s stimuli at a frequency of 10 Hz, with 4-s intervals between sequences, totaling 240 sequences (2,400 pulses). A sham stimulation followed the same protocol; however, the coil was positioned at a 90-degree angle to the scalp to ensure that the magnetic field did not penetrate the scalp. The experimental protocol is illustrated in Figure 1.

### 2.4 Data Preprocessing

All offline analyses were conducted in Matlab 2017b (MathWorks, United States) using open-source toolboxes such as EEGLAB, TMS-EEG Signal Analyzer (TESA), Fieldtrip, BrainNet viewer and Brainstorm (28, 29). Raw EEG data were epoched around TMS pulses (−1,000 to 1,000 ms), and bad channels were interpolated from adjacent channels. Fast Independent Component Analysis (FastICA) was used to remove remaining artifacts, and independent components (ICs) related to scalp muscle artifacts, ocular artifacts, and ECG artifacts were visually identified and rejected by EEG experts. The data were then re-referenced, and averages of repeated TMS stimulations were taken. The TMS-induced artifacts within a 22 ms time window (−2 to 20 ms) were replaced using the TESA toolbox with the surrounding 11 ms time windows (−13 to −2 ms and 20 to 31 ms) (30). The Cz channel, being least affected by TMS artifacts, underwent filtering using the Source-Utilized Noise Discarding (SOUND) algorithm, which removes signal components unlikely to originate from intracranial postsynaptic currents, such as electrode polarization, line noise, and electrode movement artifacts (31, 32). The data were further processed using the Signal Space Projection—Source Informed Reconstruction (SSP-SIR) algorithm to automatically eliminate residual artifacts like muscle artifacts, and a 2nd-order IIR filter was applied for 1–90 Hz band-pass filtering and 45–55 Hz notch filtering (33). FastICA was applied again to the averaged data to remove blink artifacts and suppress random noise (34). Finally, the data were re-referenced to the average reference.

### 2.5 Data analysis

After the data preprocessing, we averaged the EEG signals from all participants and experimental trials to record the TMS-EEG responses of PSCI patients before the experiment, after receiving sham stimulation, and after TMS. Time-frequency analysis was conducted using Morlet wavelet transform (with three cycles, frequency range from 1 to 90 Hz with a step size of 1 Hz, baseline correction, and a time resolution of 1–3 ms), and the results were averaged across trials and participants. In the time domain, based on previous literature (27), we identified six common peaks of TEPs: P30 (25–35 ms), N45 (35–55 ms), P60 (50–70 ms), N100 (80–120 ms), P180 (150–200 ms), and N280 (250–300 ms). The differences in TEPs within the specified time windows were analyzed, and source localization analysis was performed using Brainstorm. For the early time window (−50 to 300 ms), we compared the representative bilateral DLPFC (AF3, AF4 electrodes) across different experimental conditions. The GMFP for early TEP calculations was computed,


$$\text{GMFP}=\frac{1}{K}\sum_{i-1}^{K}V_{i}^{2}$$

where *t* is time, *V* is the voltage of channel *i*, and *K* is the number of channels (35). The experiment employed the wPLI, a functional connectivity metric that is insensitive to volume conduction or relative power changes. We calculated wPLI for different experimental conditions (Pre, Sham, and TMS) in the frequency bands of interest: theta (4–8 Hz), alpha (8–13 Hz), beta (13–30 Hz), and gamma (30–60 Hz) for AF3 with other electrodes. Subsequent statistical analyses were conducted on the wPLI for pairs of electrodes of interest, use BrainNet Viewer to map the overall connectivity before and after rTMS treatment (26).

### 2.6 Statistical analysis

The statistical analysis was conducted using SPSS 21 (IBM, New York, United States) and the FieldTrip toolbox in MATLAB 2017b. The significance level (alpha threshold) was set at 0.05 (two-tailed). Paired *t*-tests were used for data that followed a normal distribution, while the Wilcoxon signed-rank test was applied for non-normally distributed data. A paired *t*-test was conducted using Brainstorm to compare the differences in TEPs between Pre and Sham, as well as between TMS and Sham across different time windows. Additionally, the differences in wPLI for the electrodes of interest under different conditions were analyzed. Finally, the average wPLI of the theta and alpha frequency bands, which showed significant statistical differences, was correlated with cognitive scores from the MMSE and MoCA.

## 3 Results

### 3.1 Demographic and clinical characteristics

**Figure 2:**
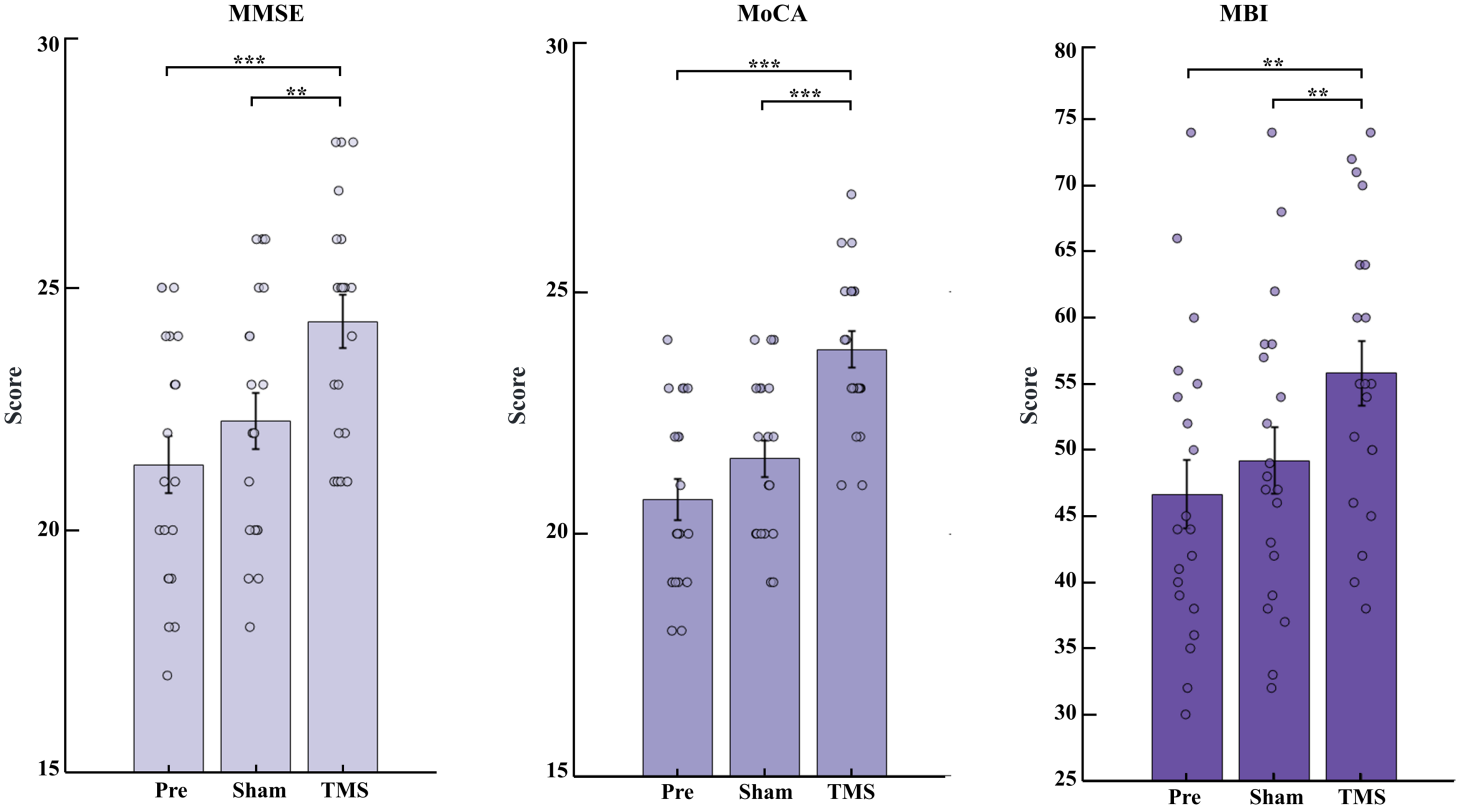
Changes in cognitive function scores and daily living abilities of PSCI patients under Pre, Sham and TMS conditions. Changes in MMSE, MoCA, and MBI scores before and after stimulation. ^**^p < 0.01 and ^***^p < 0.001.

Compared to pre-stimulation (Pre) and sham stimulation (Sham), transcranial magnetic stimulation (TMS) showed significant improvements in MMSE and MoCA scores (*p* < 0.001), and MBI scores also increased (*p* < 0.01), as shown in Figure 2.

### 3.2 TMS-EEG analysis results

**Figure 3:**
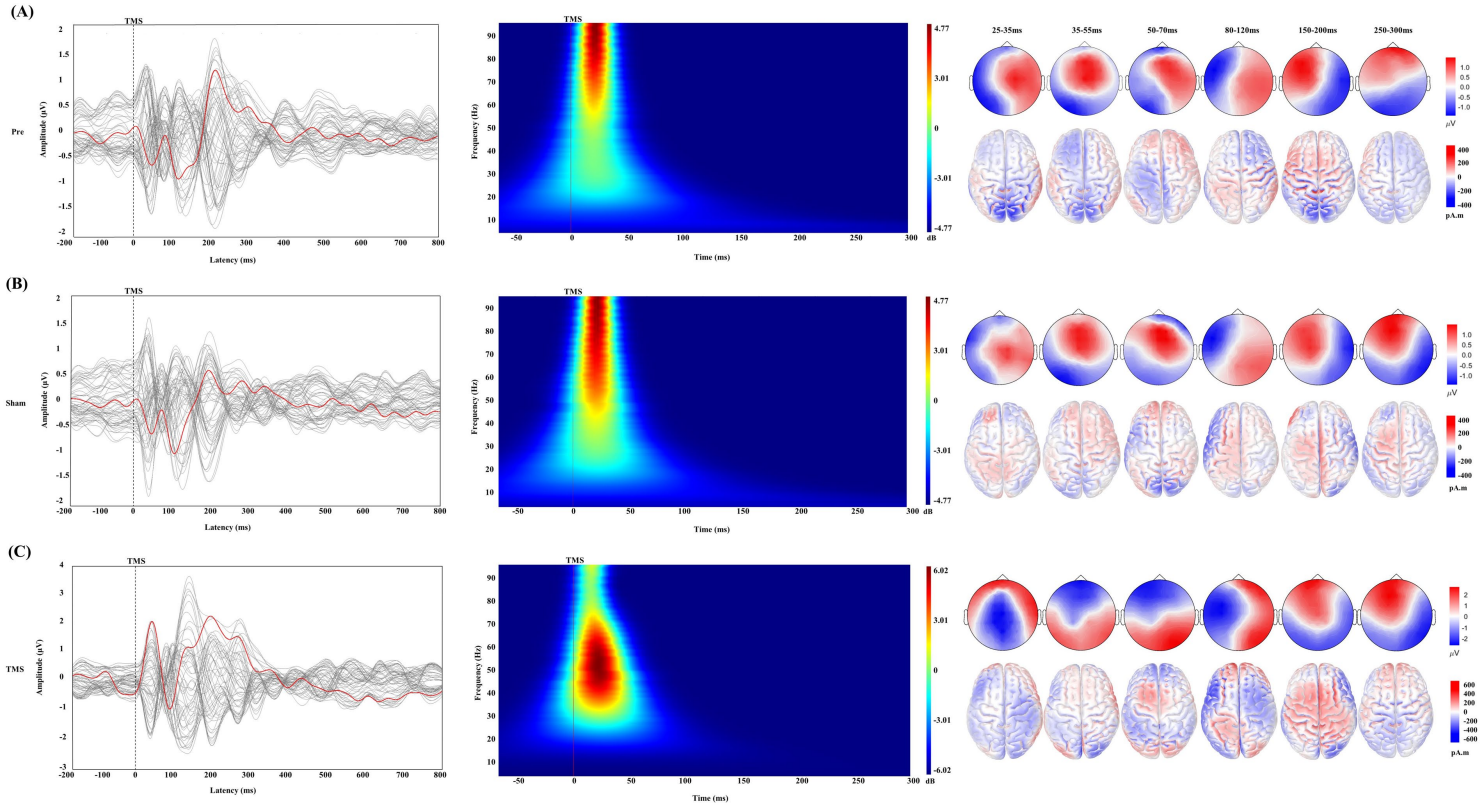
Changes in TMS-EEG in PSCI under different conditions. **(A)** Pre condition, **(B)** Sham condition, and **(C)** TMS condition. Each part corresponds to the changes in transcranial magnetic stimulation evoked potentials (TEPs), time-frequency analysis, topographical maps, and standardized low-resolution electromagnetic tomography (sLORETA) under different time windows. In the TEPs butterfly plot, the red line represents the signal from the AF3 electrode, while the gray lines represent signals from other electrodes. The gray dashed line indicates the time point of TMS stimulation. The time-frequency analysis shows the response of the AF3 electrode within the early time window (−50 to 300 ms), where the red solid line represents the time point of TMS stimulation. It also shows the changes in topographical maps and sLORETA under different time windows, with a correction bar provided on the right for better visualization.

**Figure 4:**
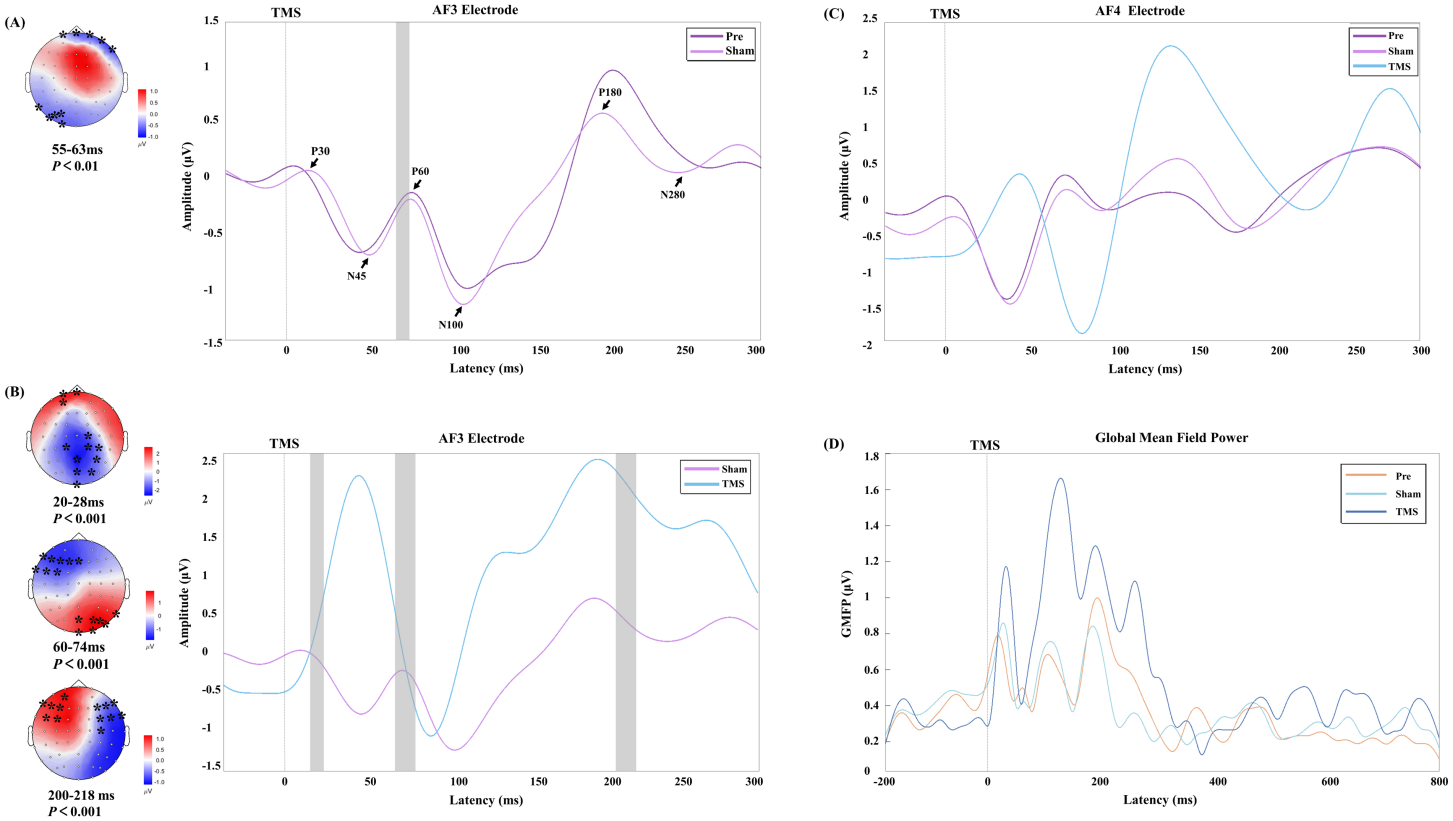
The early time window potential changes in PSCI patients under different stimulation conditions. **(A,B)** Show the time windows with significant differences at the AF3 electrode under Sham vs. Pre conditions and TMS vs. Sham conditions, respectively. The black arrows indicate the six TEP peak values under the Sham condition. In the topographic maps, the “*” symbol indicates significant clusters identified in the cluster-based permutation tests, and the gray rectangles on the right correspond to the time windows with significant differences. The color bar represents the magnitude of potential changes. **(C,D)** Present the TEP deviation patterns and changes in global mean field power (GMFP) at the AF4 electrode in PSCI patients under different conditions.

After receiving a single TMS pulse stimulation, PSCI patients exhibited greater local amplitude and slower frequency at the AF3 electrode under TMS conditions compared to the Pre and Sham conditions. Additionally, different activation patterns were observed in the topographical maps around 30 ms, 45 ms, and 60 ms (Figure 3). Therefore, we conducted an in-depth analysis of the early signals at the AF3 and AF4 electrodes within the time window of −50 to 300 ms (Figure 4).

#### 3.2.1 Plasticity effects of rTMS on TEPs

The Sham and Pre conditions showed similar deflection patterns in the bilateral DLPFC (AF3 and AF4 electrodes), with the TEP exhibiting six corresponding deflection peaks. Compared to the Sham, TMS demonstrated greater amplitude in the early time window, and the changes in the frontal and parietal-occipital regions were significant in the corresponding topographic maps.

### 3.3 The differences in connectivity of different frequency bands in PSCI patients after rTMS

**Figure 5:**
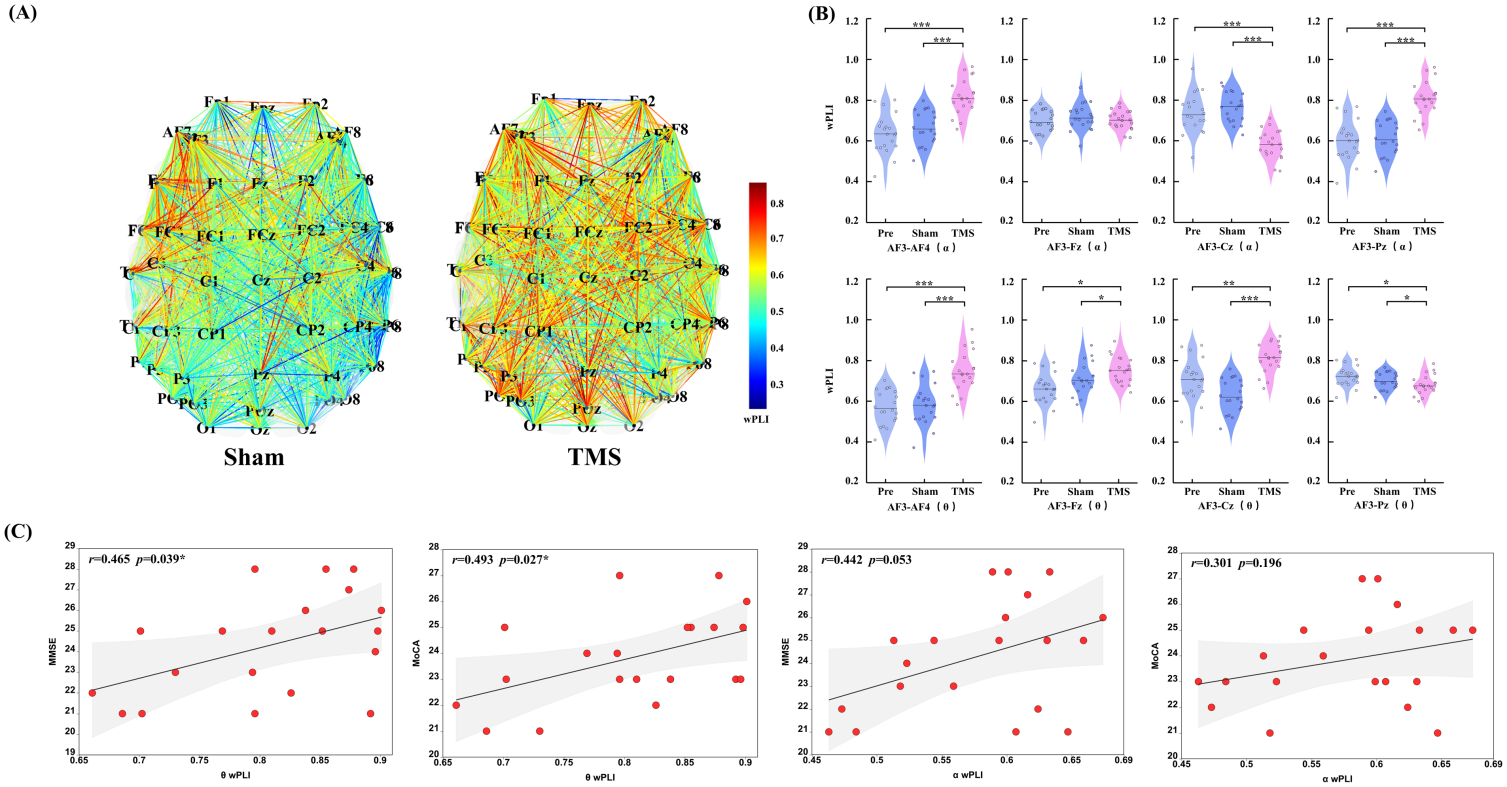
**(A)** Changes in overall functional connectivity in PSCI patients before and after rTMS treatment (under Sham and TMS conditions). **(B)** Differences in the pairs of electrodes of interest (AF3-AF4, AF3-Fz, AF3-Cz, and AF3-Pz) were analyzed in the aforementioned frequency bands and under different conditions. **(C)** An analysis was conducted on the wPLI mean values that showed significant statistical differences in the theta and alpha frequency bands, exploring their correlation with cognitive scores (MMSE and MoCA). ^*^p < 0.05, ^**^p < 0.01, and ^***^p < 0.001.

In this study, compared to the Sham group and Pre condition, PSCI patients showed significant changes in the wPLI values of AF3 with other electrodes in the theta and alpha frequency bands after TMS treatment. Further analysis of the electrodes of interest revealed that in the theta band, the connectivity between the left DLPFC and the right DLPFC and occipital region significantly increased (*p* < 0.001), while the connectivity with the central region significantly decreased (*p* < 0.001). Meanwhile, in the alpha band, the connectivity between the left DLPFC and the right DLPFC and central region significantly increased (*p* < 0.01), and the connectivity with the frontal region also significantly increased (*p* < 0.05), whereas the connectivity with the occipital region significantly decreased (*p* < 0.05). No significant differences were observed in other frequency bands and conditions. As shown in Table 2 and Figure 5B.

**Table 2:** The changes in connectivity between the AF3 electrode and other significant electrodes across different frequency bands under varying conditions.

Channel	Band	Pre	Sham	TMS
AF3-AF4	*θ*	0.57 ± 0.08	0.58 ± 0.07	0.74 ± 0.06
	*α*	0.65 ± 0.09	0.67 ± 0.10	0.81 ± 0.12
	*β*	0.42 ± 0.06	0.41 ± 0.05	0.44 ± 0.07
	*γ*	0.13 ± 0.05	0.20 ± 0.08	0.19 ± 0.09
AF3-Fz	*θ*	0.66 ± 0.10	0.70 ± 0.11	0.75 ± 0.09
	*α*	0.66 ± 0.07	0.73 ± 0.12	0.68 ± 0.08
	*β*	0.39 ± 0.05	0.40 ± 0.06	0.41 ± 0.07
	*γ*	0.19 ± 0.06	0.19 ± 0.05	0.23 ± 0.10
AF3-Cz	*θ*	0.71 ± 0.11	0.62 ± 0.09	0.82 ± 0.12
	*α*	0.73 ± 0.10	0.78 ± 0.11	0.59 ± 0.07
	*β*	0.53 ± 0.08	0.55 ± 0.09	0.52 ± 0.06
	*γ*	0.18 ± 0.05	0.17 ± 0.06	0.24 ± 0.11
AF3-Pz	*θ*	0.72 ± 0.12	0.69 ± 0.10	0.68 ± 0.08
	*α*	0.60 ± 0.07	0.61 ± 0.06	0.81 ± 0.12
	*β*	0.42 ± 0.05	0.41 ± 0.06	0.49 ± 0.07
	*γ*	0.22 ± 0.06	0.21 ± 0.05	0.25 ± 0.10

The correlation analysis between the changes in wPLI values at the AF3 electrode in the theta and alpha frequency bands and cognitive scores revealed a significant positive correlation between changes in the theta band wPLI and improvements in MMSE (*r* = 0.465, *p* = 0.039) and MoCA (*r* = 0.493, *p* = 0.027), as shown in Figure 5.

## 4 Discussion

Consistent with previous studies (17), the TEP patterns in the left DLPFC of PSCI patients were similar to those of healthy controls under both Pre and Sham conditions. However, PSCI patients exhibited higher-frequency gamma oscillations in the TEP time-frequency analysis. In comparison, rTMS induces a larger amplitude and distinct conduction pattern in the frontal region during the early TEP phase in PSCI patients, accompanied by slower gamma oscillations. Specific TEP peak characteristics are considered to reflect changes in the corresponding neurotransmitter systems. Multiple studies have shown that early TEP peaks (25–35 ms) reflect the transmission of excitatory neuro-transmitters (10), while later TEP peaks (50–70 ms) represent the balance regulation of glutamate and GABA neurotransmission (36, 37). Willis et al. (38) found that changes in glutamate and gamma-aminobutyric acid (GABA) concentrations in the temporal lobe of stroke patients are closely related to the severity of brain injury and the recovery process. A recent investigation showed that inhibitory cholinergic activity significantly enhanced the amplitude of local TEP components roughly 40 to 63 milliseconds after stimulation (39). In our study, we observed significant differences in the TEP peaks between 55–63 milli-seconds after sham stimulation in PSCI patients. Additionally, after rTMS treatment, significant differences were found between 20–28 milliseconds, 60–74 milliseconds, and 200–218 milliseconds, suggesting that these changes may be related to rTMS modulation of the corresponding neurotransmitter systems in PSCI patients. Although these findings provide electrophysiological evidence for the mechanism by which rTMS improves the cognitive function of PSCI, the specific neurotransmitter dynamics in the left DLPFC still need to be further verified through pharmacological interventions.

Xin et al. (40) found that, compared to healthy subjects, stroke patients exhibited impaired overall functional integration and reduced information transmission efficiency in their brain network connectivity. In our study, patients with PSCI exhibited a significant enhancement in the overall connectivity of brain networks following rTMS treatment. Further analysis revealed that these connectivity changes were primarily concentrated in the theta and alpha frequency bands. Although connectivity in other frequency bands also showed some degree of enhancement, no significant effects were observed. Ren et al. (41) demonstrated that the characteristics of functional connectivity in the theta band can effectively distinguish patients with mild cognitive impairment from healthy elderly individuals. Specifically, the phase-locking values (PLV) between the frontal and parieto-occipital lobes can identify cognitive impairment, providing potential biomarkers for future diagnostics. Ahmadlou et al. (42) found that the complexity of cognitive-related networks in patients with mild cognitive impairment (MCI) decreases in both alpha and theta and, with a more pronounced reduction observed in the left hemisphere in the theta band. Some studies have also found that the medial prefrontal cortex is closely related to theta activity in the implementation of cognitive control (43). Jiang et al. (44) found that MCI patients had stronger fronto-occipital and parieto-occipital functional connectivity in both the theta and alpha frequency bands. In our study, rTMS significantly altered the wPLI in the alpha and theta frequency bands in PSCI patients, enhancing the connectivity between the bilateral DLPFC. However, the changes in connectivity in the frontal regions were less pronounced compared to the bilateral DLPFC. This may be related to the early TEP conduction pattern of rTMS in PSCI patients, which is characterized by larger amplitudes in the early bilateral prefrontal regions, gradually spreading to the occipitoparietal regions, and altering the connectivity between the left DLPFC and different brain regions through neuroplasticity, thereby improving cognitive function in PSCI patients. Furthermore, we found that the wPLI in the theta frequency bands positively correlated with cognitive scores, and the changes in wPLI in the theta frequency band may serve as a potential marker for cognitive improvement in PSCI patients.

We conducted stratified subgroup analyses for key confounding variables (education level, lesion volume, and time since stroke onset) and implemented a 3-month follow-up, which revealed sustained cognitive improvement. However, the long-term effects of TMS-EEG remain unknown. Education level is one of the critical factors influencing post-stroke cognitive impairment. Numerous studies have demonstrated that patients with higher education levels tend to possess stronger cognitive reserve, thereby experiencing faster recovery of cognitive function after stroke (45). The time since stroke onset also significantly influences the occurrence of post-stroke cognitive impairment. Early intervention after stroke can markedly improve patients’ cognitive function and delay cognitive decline (46). Lesion volume is one of the important imaging characteristics associated with post-stroke cognitive impairment, as larger lesions typically result in more extensive brain damage and more severe cognitive decline (47). This study found significant differences in the impact of education level and time since stroke onset on MMSE improvement. Patients with higher education levels may benefit more from treatment due to their stronger cognitive reserve, while patients with longer time since stroke onset may face greater challenges in cognitive recovery due to reduced neuroplasticity. Regarding the minimal clinically important difference (MCID), in Alzheimer’s disease research, the MCID for the MMSE typically ranges from 1.4 to 3.75 points, while the estimated MCID for the MoCA is 1.22 or 2.15 points. However, in the PSCI population, due to the lack of data from large-scale randomized controlled trials, the MCID for cognitive scale improvements remains undefined (Reference 48: Effectiveness and Safety of the Korean Medicine Senior Health Promotion Program Using Herbal Medicine and Acupuncture for Mild Cognitive Impairment: A Retrospective Study of 500 Patients in Seoul, Korea) Table 3. However, the small sample size in this study, particularly in some subgroups, may limit the stability of the results. Future research should expand the sample size and further explore the underlying mechanisms of how education level and time since stroke onset influence the efficacy of rTMS treatment.

**Table 3:** *Post hoc* subgroup analysis of cognitive improvement in PSCI patients.

Variable	Group	*n* (%)	*β* (95% CI)	*p*	*p* for interaction
Education level (years)	All patients	20 (100%)	1.32 (0.50–2.15)	0.071	0.054
	0–6	5 (25%)	−0.58 (−1.26 to 0.10)	0.194	
	6–9	5 (25%)	−0.23 (−2.48 to 2.69)	0.862	
	>9	10 (50%)	1.28 (0.43–2.13)	0.039^*^	
Time since onset (days)	All patients	20 (100%)	2.48 (−3.37 to 8.32)	0.417	0.061
	0–30	8 (40%)	1.33 (0.07–2.59)	0.083	
	30–60	7 (35%)	0.02 (−2.59 to 2.63)	0.989	
	60–180	5 (25%)	−2.27 (−3.13 to −1.21)	0.024^*^	
Lesion size (cm^3^)	All patients	20 (100%)	0.46 (−0.69 to 1.60)	0.443	0.852
	<10	5 (25%)	0.28 (−0.09 to 0.64)	0.232	
	10–20	10 (50%)	0.63 (−0.08 to 1.34)	0.122	
	>20	5 (25%)	−0.77 (−0.59 to −0.95)	0.073	

^*^p < 0.05.

The limitations of this study include a relatively small sample size and the lack of assessment of specific cognitive domains (such as executive function, memory, and attention), which may affect the generalizability of the results. Additionally, the study only used simultaneous TMS-EEG to evaluate the effects of rTMS on cognitive function in PSCI patients, without integrating fMRI and motor evoked potentials to further assess cortical excitability and inhibitory functions. Future research plans to expand the sample size and combine multiple assessment methods to provide a more comprehensive evaluation of the impact of PSCI on different cognitive domains. Furthermore, the temporal sensitivity of TMS-EEG changes requires further investigation. Future studies will include more time points and adopt multi-session intervention designs (e.g., continuous 4 weeks or longer) to better simulate clinical practice and assess long-term effects.

## 5 Conclusion

rTMS altered the early TEP conduction patterns and functional connectivity between the left DLPFC and other brain regions in the theta and alpha frequency bands in patients with PSCI. Changes in wPLI within the theta frequency band may serve as a potential biomarker for cognitive function improvement in PSCI patients.

## Data Availability

The original contributions presented in the study are included in the article/supplementary material, further inquiries can be directed to the corresponding authors.

## References

[1] Rost NS Brodtmann A Pase MP van Veluw SJ Biffi A Duering M. Post-stroke cognitive impairment and dementia. Circ Res. (2022) 130:1252–71. doi: 10.1161/CIRCRESAHA.122.319951, PMID: 35420911

[2] Hua J Dong J Chen GC Shen Y. Trends in cognitive function before and after stroke in China. BMC Med. (2023) 21:204. doi: 10.1186/s12916-023-02908-5, PMID: 37280632 PMC10242976

[3] Asakawa T Zong L Wang L Xia Y Namba H. Unmet challenges for rehabilitation after stroke in China. Lancet. (2017) 390:121–2. doi: 10.1016/S0140-6736(17)31584-2 28699584

[4] Gao Y Qiu Y Yang Q Tang S Gong J Fan H. Repetitive transcranial magnetic stimulation combined with cognitive training for cognitive function and activities of daily living in patients with post-stroke cognitive impairment: a systematic review and meta-analysis. Ageing Res Rev. (2023) 87:101919. doi: 10.1016/j.arr.2023.101919, PMID: 37004840

[5] Han K Liu J Tang Z Su W Liu Y Lu H. Effects of excitatory transcranial magnetic stimulation over the different cerebral hemispheres dorsolateral prefrontal cortex for post-stroke cognitive impairment: a systematic review and meta-analysis. Front Neurosci. (2023) 17:1102311. doi: 10.3389/fnins.2023.1102311, PMID: 37260845 PMC10228699

[6] Salavati B Daskalakis ZJ Zomorrodi R Blumberger DM Chen R Pollock BG. Pharmacological modulation of long-term potentiation-like activity in the dorsolateral prefrontal cortex. Front Hum Neurosci. (2018) 12:155. doi: 10.3389/fnhum.2018.00155, PMID: 29740299 PMC5928132

[7] Lioumis P Rosanova M. The role of neuronavigation in TMS-EEG studies: current applications and future perspectives. J Neurosci Methods. (2022) 380:109677. doi: 10.1016/j.jneumeth.2022.109677, PMID: 35872153

[8] Casali AG Casarotto S Rosanova M Mariotti M Massimini M. General indices to characterize the electrical response of the cerebral cortex to TMS. NeuroImage. (2010) 49:1459–68. doi: 10.1016/j.neuroimage.2009.09.026, PMID: 19770048

[9] Hadiyoso S Zakaria H Mengko TLER Ong PA. (2021). Preliminary study of EEG characterization using power spectral analysis in post-stroke patients with cognitive impairment. Proceedings of the 1st International Conference on Electronics, Biomedical Engineering, and Health Informatics

[10] Ferreri F Pasqualetti P Määttä S Ponzo D Ferrarelli F Tononi G. Human brain connectivity during single and paired pulse transcranial magnetic stimulation. NeuroImage. (2011) 54:90–102. doi: 10.1016/j.neuroimage.2010.07.056, PMID: 20682352

[11] Kallioniemi E Daskalakis ZJ. Identifying novel biomarkers with TMS-EEG - methodological possibilities and challenges. J Neurosci Methods. (2022) 377:109631. doi: 10.1016/j.jneumeth.2022.109631, PMID: 35623474

[12] Dang G Su X Yang M Che S Ren H Li Z. Abnormal brain functional connectivity after subcortical stroke: a TMS-EEG study. Brain Stimul. (2019) 12:569. doi: 10.1016/j.brs.2018.12.886, PMID: 40258974

[13] Keser Z Buchl SC Seven NA Markota M Clark HM Jones DT. Electroencephalogram (EEG) with or without transcranial magnetic stimulation (TMS) as biomarkers for post-stroke recovery: a narrative review. Front Neurol. (2022) 13:827866. doi: 10.3389/fneur.2022.827866, PMID: 35273559 PMC8902309

[14] Kan R Padberg F Giron CG Lin T Zhang B Brunoni AR. Effects of repetitive transcranial magnetic stimulation of the left dorsolateral prefrontal cortex on symptom domains in neuropsychiatric disorders: a systematic review and cross-diagnostic meta-analysis. Lancet Psychiatry. (2023) 10:252–9. doi: 10.1016/S2215-0366(23)00026-3, PMID: 36898403

[15] Liu M Bao G Bai L Yu E. The role of repetitive transcranial magnetic stimulation in the treatment of cognitive impairment in stroke patients: a systematic review and meta-analysis. Sci Prog. (2021) 104:368504211004266. doi: 10.1177/00368504211004266, PMID: 33827345 PMC10455033

[16] Chen X Liu F Lyu Z Xiu H Hou Y Tu S. High-frequency repetitive transcranial magnetic stimulation (HF-rTMS) impacts activities of daily living of patients with post-stroke cognitive impairment: a systematic review and meta-analysis. Neurol Sci. (2023) 44:2699–713. doi: 10.1007/s10072-023-06779-9, PMID: 37012519

[17] Rogasch NC Daskalakis ZJ Fitzgerald PB. Cortical inhibition of distinct mechanisms in the dorsolateral prefrontal cortex is related to working memory performance: a TMS-EEG study. Cortex. (2015) 64:68–77. doi: 10.1016/j.cortex.2014.10.003, PMID: 25461708

[18] Nasreddine ZS Phillips NA Bédirian V Charbonneau S Whitehead V Collin I. The Montreal Cognitive Assessment, MoCA: a brief screening tool for mild cognitive impairment. J Am Geriatr Soc. (2005) 53:695–9. doi: 10.1111/j.1532-5415.2005.53221.x, PMID: 15817019

[19] Katzman R Zhang MY Ouang-Ya-Qu Wang ZY Liu WT Yu E. A Chinese version of the Mini-Mental State Examination; impact of illiteracy in a Shanghai dementia survey. J Clin Epidemiol. (1988) 41:971–8. doi: 10.1016/0895-4356(88)90034-0, PMID: 3193141

[20] Tang Y. The MoCA as a cognitive screening tool for mild cognitive impairment (MCI) in elderly adults in China. Psychiatry Res. (2020) 291:113210. doi: 10.1016/j.psychres.2020.113210, PMID: 32540686

[21] Kleindorfer DO Towfighi A Chaturvedi S Cockroft KM Gutierrez J Lombardi-Hill D. 2021 guideline for the prevention of stroke in patients with stroke and transient ischemic attack: a guideline from the American Heart Association/American Stroke Association. Stroke. (2021) 52:e364–467. doi: 10.1161/STR.0000000000000375, PMID: 34024117

[22] Shimizu T Hosaki A Hino T Sato M Komori T Hirai S. Motor cortical disinhibition in the unaffected hemisphere after unilateral cortical stroke. Brain. (2002) 125:1896–907. doi: 10.1093/brain/awf183, PMID: 12135979

[23] Rossini PM Barker AT Berardelli A Caramia MD Caruso G Cracco RQ. Non-invasive electrical and magnetic stimulation of the brain, spinal cord and roots: basic principles and procedures for routine clinical application. Report of an IFCN committee. Electroencephalogr Clin Neurophysiol. (1994) 91:79–92. doi: 10.1016/0013-4694(94)90029-9, PMID: 7519144

[24] Pennisi G Rapisarda G Bella R Calabrese V Maertens De Noordhout A Delwaide PJ. Absence of response to early transcranial magnetic stimulation in ischemic stroke patients: prognostic value for hand motor recovery. Stroke. (1999) 30:2666–70. doi: 10.1161/01.str.30.12.2666, PMID: 10582994

[25] Veldema J Nowak DA Gharabaghi A. Resting motor threshold in the course of hand motor recovery after stroke: a systematic review. J Neuroeng Rehabil. (2021) 18:158. doi: 10.1186/s12984-021-00947-8, PMID: 34732203 PMC8564987

[26] Bai Z Zhang JJ Fong K. Intracortical and intercortical networks in patients after stroke: a concurrent TMS-EEG study. J Neuroeng Rehabil. (2023) 20:100. doi: 10.1186/s12984-023-01223-7, PMID: 37533093 PMC10398934

[27] Tremblay S Rogasch NC Premoli I Blumberger DM Casarotto S Chen R. Clinical utility and prospective of TMS-EEG. Clin Neurophysiol. (2019) 130:802–44. doi: 10.1016/j.clinph.2019.01.001, PMID: 30772238

[28] Medani T Garcia-Prieto J Tadel F Antonakakis M Erdbrügger T Höltershinken M. Brainstorm-DUNEuro: an integrated and user-friendly finite element method for modeling electromagnetic brain activity. NeuroImage. (2023) 267:119851. doi: 10.1016/j.neuroimage.2022.119851, PMID: 36599389 PMC9904282

[29] Xia M Wang J He Y. BrainNet viewer: a network visualization tool for human brain connectomics. PLoS One. (2013) 8:e68910. doi: 10.1371/journal.pone.0068910, PMID: 23861951 PMC3701683

[30] Rogasch NC Sullivan C Thomson RH Rose NS Bailey NW Fitzgerald PB. Analysing concurrent transcranial magnetic stimulation and electroencephalographic data: a review and introduction to the open-source TESA software. NeuroImage. (2017) 147:934–51. doi: 10.1016/j.neuroimage.2016.10.031, PMID: 27771347

[31] Mutanen TP Kukkonen M Nieminen JO Stenroos M Sarvas J Ilmoniemi RJ. Recovering TMS-evoked EEG responses masked by muscle artifacts. NeuroImage. (2016) 139:157–66. doi: 10.1016/j.neuroimage.2016.05.028, PMID: 27291496

[32] Mutanen TP Biabani M Sarvas J Ilmoniemi RJ Rogasch NC. Source-based artifact-rejection techniques available in TESA, an open-source TMS-EEG toolbox. Brain Stimul. (2020) 13:1349–51. doi: 10.1016/j.brs.2020.06.079, PMID: 32659484

[33] Uusitalo MA Ilmoniemi RJ. Signal-space projection method for separating MEG or EEG into components. Med Biol Eng Comput. (1997) 35:135–40. doi: 10.1007/BF02534144 9136207

[34] Hyvärinen A. Fast and robust fixed-point algorithms for independent component analysis. IEEE Trans Neural Netw. (1999) 10:626–34. doi: 10.1109/72.761722, PMID: 18252563

[35] Lehmann D Skrandies W. Reference-free identification of components of checkerboard-evoked multichannel potential fields. Electroencephalogr Clin Neurophysiol. (1980) 48:609–21. doi: 10.1016/0013-4694(80)90419-8, PMID: 6155251

[36] Belardinelli P König F Liang C Premoli I Desideri D Müller-Dahlhaus F. TMS-EEG signatures of glutamatergic neurotransmission in human cortex. Sci Rep. (2021) 11:8159. doi: 10.1038/s41598-021-87533-z, PMID: 33854132 PMC8047018

[37] Premoli I Castellanos N Rivolta D Belardinelli P Bajo R Zipser C. TMS-EEG signatures of GABAergic neurotransmission in the human cortex. J Neurosci. (2014) 34:5603–12. doi: 10.1523/JNEUROSCI.5089-13.2014, PMID: 24741050 PMC6608220

[38] Willis HE Ip IB Watt A Campbell J Jbabdi S Clarke WT. GABA and glutamate in hMT+ link to individual differences in residual visual function after occipital stroke. Stroke. (2023) 54:2286–95. doi: 10.1161/STROKEAHA.123.043269, PMID: 37477008 PMC10453332

[39] Song Y Gordon PC Roy O Metsomaa J Belardinelli P Rostami M. Involvement of muscarinic acetylcholine receptor-mediated cholinergic neurotransmission in TMS-EEG responses. Prog Neuro-Psychopharmacol Biol Psychiatry. (2025) 136:111167. doi: 10.1016/j.pnpbp.2024.111167, PMID: 39383933

[40] Xin X Duan F Kranz GS Shu D Fan R Gao Y. Functional network characteristics based on EEG of patients in acute ischemic stroke: a pilot study. NeuroRehabilitation. (2022) 51:455–65. doi: 10.3233/NRE-220107, PMID: 35848041

[41] Ren Z Zhao Y Han X Yue M Wang B Zhao Z. An objective model for diagnosing comorbid cognitive impairment in patients with epilepsy based on the clinical-EEG functional connectivity features. Front Neurosci. (2022) 16:1060814. doi: 10.3389/fnins.2022.1060814, PMID: 36711136 PMC9878185

[42] Ahmadlou M Adeli A Bajo R Adeli H. Complexity of functional connectivity networks in mild cognitive impairment subjects during a working memory task. Clin Neurophysiol. (2014) 125:694–702. doi: 10.1016/j.clinph.2013.08.033, PMID: 24405905

[43] Cavanagh JF Frank MJ. Frontal theta as a mechanism for cognitive control. Trends Cogn Sci. (2014) 18:414–21. doi: 10.1016/j.tics.2014.04.012, PMID: 24835663 PMC4112145

[44] Jiang Y Zhang X Guo Z Jiang N. Altered EEG theta and alpha band functional connectivity in mild cognitive impairment during working memory coding. IEEE Trans Neural Syst Rehabil Eng. (2024) 32:2845–53. doi: 10.1109/TNSRE.2024.3417617, PMID: 38905095

[45] Shin M Sohn MK Lee J Kim DY Lee SG Shin YI. Effect of cognitive reserve on risk of cognitive impairment and recovery after stroke: the KOSCO study. Stroke. (2020) 51:99–107. doi: 10.1161/STROKEAHA.119.026829, PMID: 31822247 PMC6924936

[46] Hurford R Charidimou A Fox Z Cipolotti L Werring DJ. Domain-specific trends in cognitive impairment after acute ischaemic stroke. J Neurol. (2013) 260:237–41. doi: 10.1007/s00415-012-6625-0, PMID: 22865200

[47] Lim JS Lee JJ Woo CW. Post-stroke cognitive impairment: pathophysiological insights into brain disconnectome from advanced neuroimaging analysis techniques. J Stroke. (2021) 23:297–311. doi: 10.5853/jos.2021.02376, PMID: 34649376 PMC8521255

